# Causal impact of human blood metabolites and metabolic pathways on serum uric acid and gout: a mendelian randomization study

**DOI:** 10.3389/fendo.2024.1378645

**Published:** 2024-07-04

**Authors:** Yan Zhong, ChengAn Yang, BingHua Zhang, YingWen Chen, WenJun Cai, GuoChen Wang, ChangWei Zhao, WenHai Zhao

**Affiliations:** ^1^ College of Traditional Chinese Medicine, Changchun University of Chinese Medicine, Changchun, China; ^2^ College of Integrated Chinese and Western Medicine, Tianjin University of Chinese Medicine, Tianjin, China; ^3^ Department of Orthopaedic Center, The Third Clinical Hospital of Changchun University of Chinese Medicine, Changchun, China; ^4^ Department of Orthopedics Center, Affiliated Hospital of Changchun University of Chinese Medicine, Changchun, China

**Keywords:** blood metabolite, Mendelian randomization, hyperuricemia, gout, risk factor

## Abstract

**Objective:**

Hyperuricaemia and gout are common metabolic disorders. However, the causal relationships between blood metabolites and serum urate levels, as well as gout, remain unclear. A systematic evaluation of the causal connections between blood metabolites, hyperuricemia, and gout could enhance early screening and prevention of hyperuricemia and gout in clinical settings, providing novel insights and approaches for clinical treatment.

**Methods:**

In this study, we employed a bidirectional two-sample Mendelian randomization analysis utilizing data from a genome-wide association study involving 7,286 participants, encompassing 486 blood metabolites. Serum urate and gout data were sourced from the Chronic Kidney Disease Genetics consortium, including 288,649 participants for serum urate and 9,819 African American and 753,994 European individuals for gout. Initially, LDSC methodology was applied to identify blood metabolites with a genetic relationship to serum urate and gout. Subsequently, inverse-variance weighting was employed as the primary analysis method, with a series of sensitivity and pleiotropy analyses conducted to assess the robustness of the results.

**Results:**

Following LDSC, 133 blood metabolites exhibited a potential genetic relationship with serum urate and gout. In the primary Mendelian randomization analysis using inverse-variance weighting, 19 blood metabolites were recognized as potentially influencing serum urate levels and gout. Subsequently, the IVW p-values of potential metabolites were corrected using the false discovery rate method. We find leucine (IVW P _FDR_ = 0.00004), N-acetylornithine (IVW P _FDR_ = 0.0295), N1-methyl-3-pyridone-4-carboxamide (IVW P _FDR_ = 0.0295), and succinyl carnitine (IVW P _FDR_ = 0.00004) were identified as significant risk factors for elevated serum urate levels. Additionally, 1-oleoylglycerol (IVW P _FDR_ = 0.0007) may lead to a substantial increase in the risk of gout. Succinyl carnitine exhibited acceptable weak heterogeneity, and the results for other blood metabolites remained robust after sensitivity, heterogeneity, and pleiotropy testing. We conducted an enrichment analysis on potential blood metabolites, followed by a metabolic pathway analysis revealing four pathways associated with serum urate levels.

**Conclusion:**

The identified causal relationships between these metabolites and serum urate and gout offer a novel perspective, providing new mechanistic insights into serum urate levels and gout.

## 1 Introduction

Hyperuricaemia stands as the primary risk factor for gout, playing a central role in its pathogenesis. The primary routes for urate excretion involve the kidneys, intestine, and liver, with impaired renal excretion as the principal cause of hyperuricaemia. Saturation of SU results in the deposition of monocrystalline sodium urate (MSU) in the joints (1). Substantial MSU deposition leads to the formation of gout stones, causing additional damage to joint cartilage. Prolonged hyperuricaemia contributes to both renal function decline and kidney inflammation (2). Effective prevention and treatment are key to reducing hyperuricaemia incidence. Understanding the biological mechanisms of hyperuricaemia is crucial for preventing and treating gout. Despite significant contributions from genetic studies, the development of hyperuricaemia and gout remains a complex process (3). Recent evidence indicates a close relationship between SU levels, gout, and metabolic abnormalities. Widely recognized, hyperuricaemia is a common complication in individuals with metabolic syndrome (4). Individuals with insulin resistance face an elevated risk of developing hyperuricaemia, linked to the well-established, long-standing negative correlation between insulin resistance and renal clearance. Specifically, a reduction in renal clearance increases susceptibility to hyperuricaemia (5). Weight loss measures, particularly for those with visceral obesity, can decrease susceptibility to hyperuricaemia (6). Previous research has shown that hyperuricemia may increase the risk of cardiovascular disease by causing endothelial dysfunction through oxidative stress (7). Specifically, serum uric acid (SUA) production is closely linked with xanthine oxidoreductase (XOR). The oxidants produced by XOR activation can oxidize low-density lipoprotein (LDL), leading to the activation of inflammasomes and atherosclerosis, thus increasing the risk of cardiovascular disease (8). In this context, urate-lowering therapy appears to be a feasible treatment option for hyperuricemia with concomitant cardiovascular disease (9). Lowering uric acid levels is considered a method to optimize blood pressure regulation (10). Additionally, urate-lowering treatment with allopurinol can inhibit the renal reabsorption of uric acid, providing protection to the kidneys (11).

Gout stands as the most prevalent global form of inflammatory arthritis, primarily triggered by a sustained increase in uric acid levels, resulting in hyperuricemia and ultimately leading to the onset of gout (12). Clinical manifestations encompass intense pain in the joints of the lower limbs, primarily attributed to the deposition of MSU, intricately linked with both patient mortality and morbidity rates (13). As gout progresses, some individuals may experience advanced symptoms, characterized by persistent chronic pain and the formation of gout stones. The incidence of gout has steadily risen since the 20th century, possibly influenced by shifts in population age demographics, metabolic syndrome, and environmental factors. Reports from Europe and North America reveal an adult gout prevalence ranging from 0.68% to 3.9%, featuring gender disparities in both incidence and mortality, favoring males at a ratio of approximately 2:1 (14–17). Presently, treatment options for gout patients are severely limited, with less than one-third undergoing treatment and fewer than half adhering to prescribed regimens. This limitation has contributed to increased hospitalization and disability rates, resulting in diminished social productivity for both patients and their families, along with a heightened economic burden (18).

Researchers have diligently investigated the mechanisms underlying the inflammatory response triggered by MSU. However, persistent questions remain, such as the variability in patients developing either acute or chronic gout. The specific triggers for gout attacks and the factors preventing most hyperuricemia patients from transitioning to gout remain elusive. Consequently, there is an urgent need for exploring potential biomarkers indicating elevated SU levels and the initiation of gout. This represents a critical area that requires focused investigation.

Long-term metabolic disruptions lay the groundwork for the onset of hyperuricemia and gout, prompting an intensified examination of the roles played by metabolomics and immunomics in unraveling the intricate pathogenic mechanisms and physiological-pathological changes associated with this condition. Researchers have unearthed a correlation between hyperuricemia and gout not only with uric acid but also with other blood metabolites, prompting a novel avenue of exploration (19). Currently, blood metabolites exhibit substantial advantages in disease diagnosis and prognosis, offering fresh insights into predicting both hyperuricemia and gout (20). Thus, blood metabolites assume a pivotal role in anticipating the development of gout and uric acid level. However, the relationship between blood metabolites, SU, and gout remains unclear due to limitations in sample size and interference from confounding factors.

As a novel and robust epidemiological research approach, MR analysis employs genetic variations as instrumental variables to investigate causal relationships between exposure and outcomes. The genetic type is determined at conception, eliminating interference from external confounding factors and allowing MR studies to effectively minimize bias. Recent extensions of MR studies into metabolomics necessitate (21–24) assessing whether causal relationships exist between blood metabolites, SU, and gout, shedding light on the roles of blood metabolites in the disease. The objectives of this study involve employing various MR analysis methods to identify blood metabolites influencing SU levels and gout, examining the reciprocal impact of SU levels and gout on blood metabolites, and elucidating potential metabolic pathways affecting SU levels and gout. These insights aim to contribute novel perspectives for early diagnosis and prevention.

## 2 Research design

**Figure 1:**
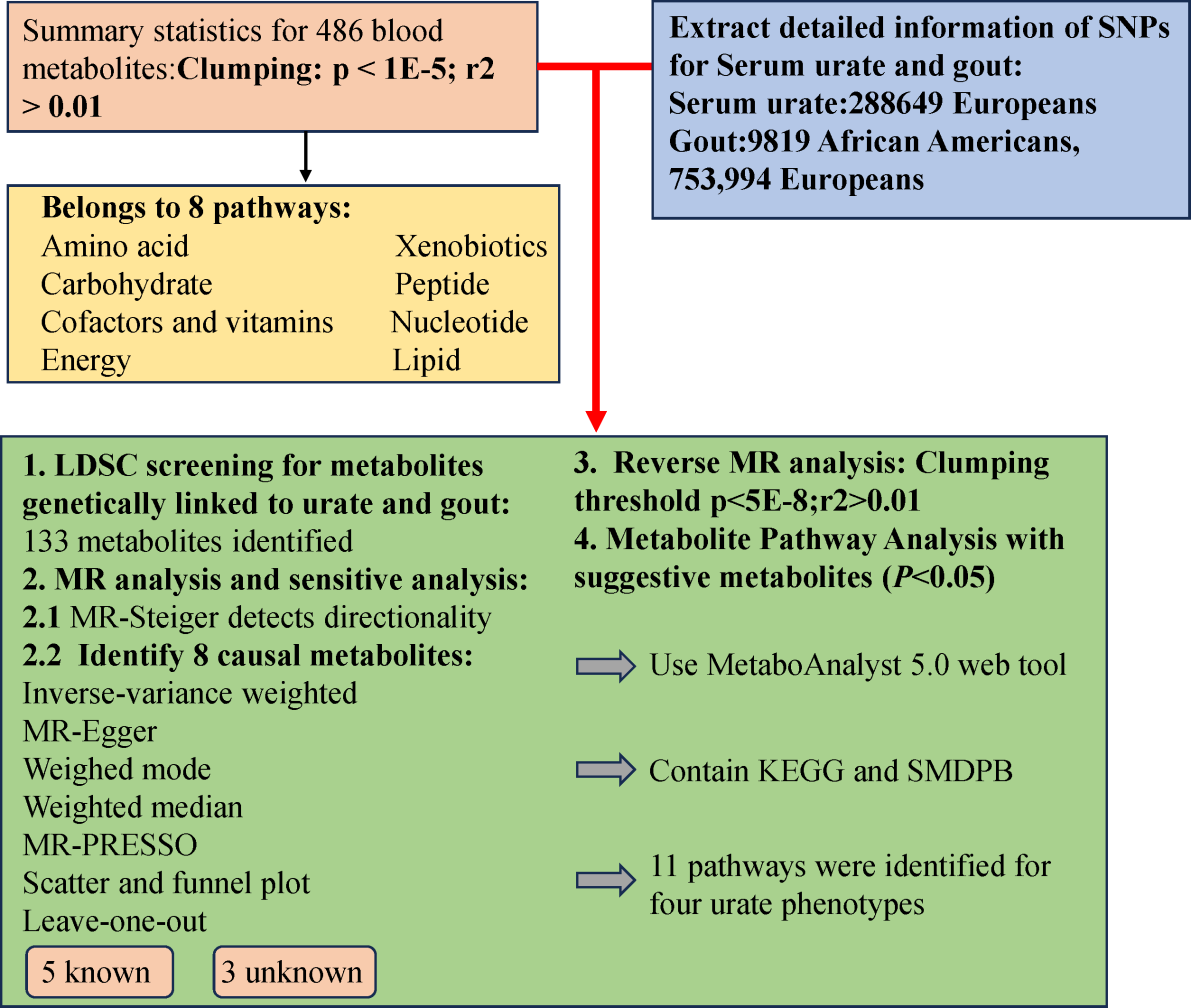
Mendelian randomization research process.

The study design involved several crucial steps. Initially, we employed Linkage Disequilibrium Score Regression (LDSC) analysis to assess the genetic correlation using 486 blood metabolites as exposures, and SU and gout as outcomes. Subsequently, a comprehensive evaluation was conducted to determine the causal relationships between blood metabolites exhibiting potential genetic correlations and the risks of SU and gout. To ensure the rigor of the MR study, three conditions were met: (1) Instrumental Variables (IVs) were directly related to exposure; (2) IVs were unrelated to the outcome and remained unaffected by confounding factors; (3) IVs influenced outcomes solely through exposure. Importantly, data on blood metabolites, SU, and gout were sourced from independent Genome-Wide Association Study (GWAS) datasets to prevent sample overlap. The research methodology adhered to the STROBE-MR checklist (25), and a visual representation of the study design is provided in **Figure 1**.

### 2.1 Human blood metabolites GWAS dataset

The study harnessed a comprehensive GWAS dataset encompassing 486 human blood metabolites, as derived from Shin et al.’s research (26). Accessible through the Metabolomics GWAS database (http://metabolomics.helmholtz-muenchen.de/gwas/), this dataset was meticulously curated, with Shin and colleagues meticulously measuring the 486 blood metabolites within a European cohort of 7824 individuals. Notably, the dataset comprises data from two primary studies: the German KORA F4 study (1768 participants) and the British Twin study (6056 participants). Ethical approval was diligently obtained from local ethics committees before the initiation of both studies, and active informed consent was secured from all participants. Out of the entire spectrum of blood metabolites, 309 are identified, while 309 blood metabolites are identified, the classification of the rest as ‘unknown’ is attributed to the ambiguity of their chemical properties. Researchers further categorized the known blood metabolites into 8 distinct metabolic classes, aligning with the Kyoto Encyclopedia of Genes and Genomes (KEGG) database definition (27).

### 2.2 GWAS dataset on serum urate and gout

The study relies on extensive data from the Chronic Kidney Disease Genetics Consortium (CKDGen), representing the most comprehensive GWAS datasets for SU and gout to date. These datasets include SU data from 288,649 individuals and encompass 763,813 gout patients of European descent, consisting of 9,819 African Americans and 753,994 Europeans (28). Detailed information about the datasets is available in the initial GWAS, conducted with the informed consent of all participants. Definitions for SU and gout include self-reports, hospital diagnoses, and International Classification of Diseases, Tenth Edition (ICD-10) codes. Rigorous data curation involved excluding SNPs with non-biallelic genotypes, lacking rsIDs, and mismatching bases or alleles with the 10,000 genomes. Given our study focus on SU and gout outcomes and the presence of urate data in the blood metabolites dataset, we opted to exclude urate data from the latter for a more focused analysis.

### 2.3 LDSC regression

LDSC regression analysis, a novel method for evaluating SNP heritability, assesses GWAS summary data instead of individual-level data (29). Common confounding biases and polygenic effects significantly contribute to the inflation of statistical quantities. LDSC corrects for polygenic effects in GWAS analysis, enhancing the detection of the regression relationship (r2) between statistical quantities and LD. It quantifies the entropy of each variable and employs its intercept value to distinguish and correct the causes of statistical inflation (30). Therefore, our initial LDSC analysis aimed to explore potential genetic relationships between blood metabolites and SU, as well as gout. We considered a potential genetic relationship when rg-p<0.05, enabling us to not only assess genetic patterns between the two but also evaluate potential genetic associations.

### 2.4 Selection of instrumental variables

Due to the limited samples of blood metabolites, we employed a series of methods for a more refined screening of eligible instrumental variables. Initially, we selected a threshold of P<1×10^-5^, referencing 1000 genome projects, and set the linkage disequilibrium (LD) threshold r^2^<0.001 within a 500 bases pair KB distance. This approach has been extensively utilized in prior MR studies (22, 23, 31). Simultaneously, we computed the F-statistic for each SNP, excluding those with F<10 as weak instrumental variables to ensure adequate statistical power for all blood metabolites (27).

### 2.5 MR analysis

Firstly, due to observed bidirectional associations, we conducted the MR-Steiger test to ensure that the results we observed are not influenced by reverse causation. The initial analysis used the standard inverse variance-weighted (IVW) method for assessment, which is often more accurate when the selected instrumental variables meet the three assumptions of MR analysis (32). However, if some instrumental variables contradict the assumptions of MR, the analysis results may be inaccurate. Therefore, we introduced secondary methods for further analysis: 1. Employing Cochran’s Q test to ensure no heterogeneity among individual IVW results. 2. Addressing potential horizontal pleiotropy, we utilized the MR-PRESSO method to detect outliers and assess possible horizontal pleiotropy. Additionally, we applied the MR-Egger intercept test to estimate intercept-based pleiotropy and detect genetic variation independent of exposure and outcome (33). 3. Conducting additional analyses using two different hypothesis methods (weighted median and weighted mode) to enhance the robustness of the MR study (34). Although the accuracy of weighted median and MR-Egger as secondary tests may not match IVW, results are often reliable when these three approaches align (35). Finally, we adopted the “leave-one-out” method to evaluate if individual SNPs drive MR outcomes.

### 2.6 Reverse MR analysis

To investigate the potential causal relationship between SU, gout, and previously identified correlated blood metabolites, we conducted a reverse MR study. In this study, genetic variants associated with SU and gout were employed as Instrumental variable, while confirmed blood metabolites were designated as outcomes.

### 2.7 Metabolic pathway analysis

Adopting metaconconflict 5.0 (https://www.metaboanalyst.ca/) (36), we conducted a pathway analysis of the finalized blood metabolites based on KEGG and metabolic pathways. This user-friendly bioinformatics website simplifies the process of metabolomics analysis, providing valuable insights into the metabolic pathways associated with the identified blood metabolites.

### 2.8 Statistical analysis

Statistical analyses were carried out using R 4.2.3 software. Initially, the “LDSCr” package was employed for assessment, followed by Mendelian randomization analysis using the “TwoSampleMR” MR package. To control potential “false positives” false discovery rate (FDR) correction was applied, and causality was considered significant at a P_FDR_ < 0.05.

## 3 Results

After LDSC analysis, we identified potential correlations between 102 blood metabolites and SU, as well as 32 blood metabolites potential correlated with gout (**Supplementary File, Table** **S1**).

The number of SNPs in IV varies from 2 to 180. All SNPs showed F-value statistics exceeding 10, indicating the absence of weak instrumental variables (**Supplementary File, Table S2**).

### 3.1 Preliminary analysis

Under the consistent directionality of the three methods, a preliminary examination of 102 genetically related blood metabolites to SU revealed 16 with potential impacts detected through the IVW method (IVW, p < 0.05). These include 8 in amino acid metabolism, 2 in lipid metabolism, 2 in nucleotide metabolism, 1 in energy metabolism, and 4 in unknown metabolism. Among known blood metabolites, 4 are linked to a reduced SU risk: glutamine (β= -0.79; 95% CI -1.56, -0.02; P= 0.044), 3-methyl-2-oxovalerate (β= -0.24; 95% CI -0.42, -0.06; P= 0.01), 1-heptadecanoylglycerophosphocholine (β= -0.24; 95% CI -0.42, -0.06; P= 0.033), and tryptophan betaine (β= -0.05; 95% CI -0.09, -0.01; P= 0.006). Eight blood metabolites are associated with an increased SU, including leucine (β= 0.35; 95% CI 0.21, 0.49; P=<0.001), 5-oxoproline (β= 0.18; 95% CI 0.002, 0.36; P= 0.046), carnitine (β= 0.15; 95% CI 0.02, 0.29; P= 0.022), N-acetylornithine (β= 1.08; 95% CI 1.03-1.13; P= 0.002), alanine (β= 0.2; 95% CI 0.04, 0.36; P= 0.015), N1-methyl-3-pyridone-4-carboxamide (β= 0.17; 95% CI 0.06, 0.28; P= 0.002), N2-dimethylguanosine (β= 0.12; 95% CI 0.01, 0.23; P= 0.032), and succinyl carnitine (β= 0.22; 95% CI 0.09, 0.36; P= 0.001) (**Supplementary File, Table S3**). Additionally, we conducted MR-Steiger directional testing on 16 blood metabolites related to SU and 3 blood metabolites related to gout. The results showed no directional abnormalities when blood metabolites were used as exposure, and SU and gout were considered as outcomes (**Supplementary File, Table S4**).

**Figure 2:**
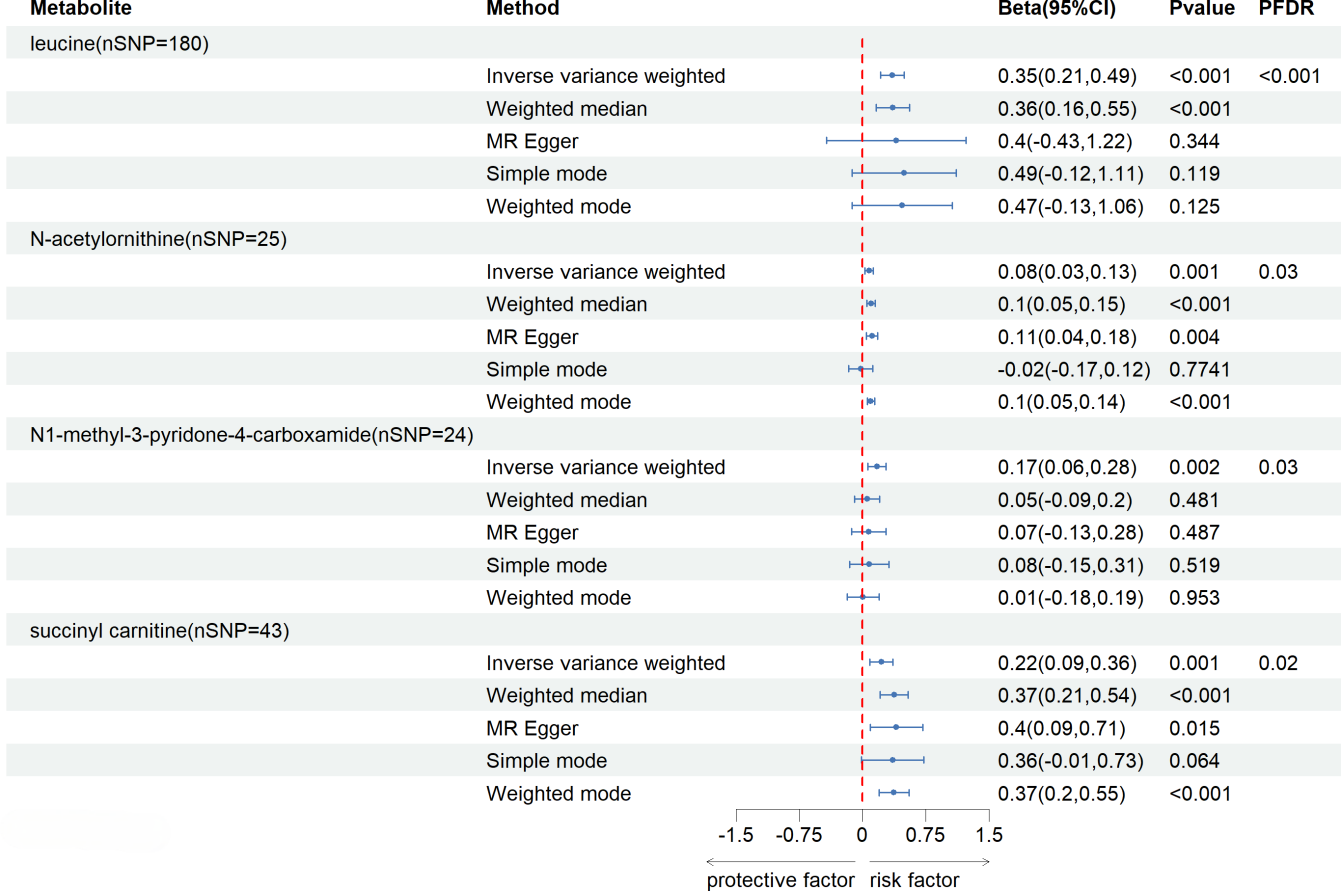
Known Blood metabolites Showing a Significant Relationship with SU After FDR. CI, confidence interval; Beta(95%CI), 95% confidence interval for beta; PDR, P-value for error rate correction.

**Figure 3:**
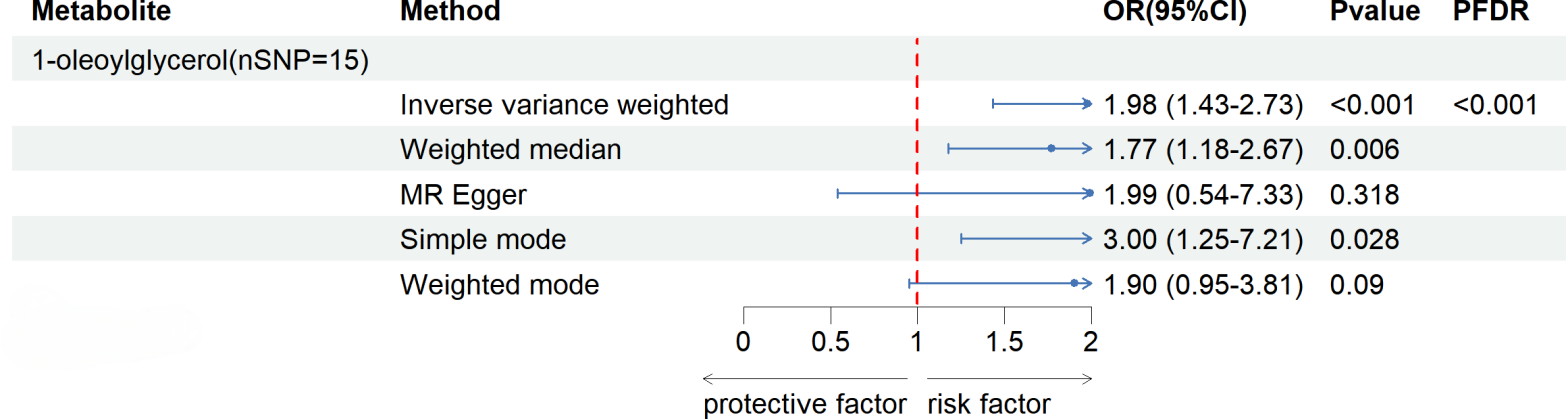
Known Blood metabolites Showing a Significant Relationship with gout After FDR Correctiontics for IVW. Correction. OR, odds ratio; OR (95%CI), 95% confidence interval for OR.

After FDR correction (FDR < 0.05), five blood metabolites exhibited a significant correlation, with one being an unidentified metabolite. Among the four known significantly correlated blood metabolites, two were associated with amino acid metabolism, one with energy metabolism, and one with nucleotide metabolism. These blood metabolites are identified as risk factors for elevated SU levels: leucine (P _FDR_ = 0.00004), N-acetylornithine (P _FDR_ = 0.0295), N1-methyl-3-pyridone-4-carboxamide (P _FDR_ = 0.0295), and succinyl carnitine (P _FDR_ = 0.00004) (**Figure 2**). Of the 31 blood metabolites associated with gout after LDSC screening, only 1-oleoylglycerol (OR = 1.98; 95% CI 1.43-2.73, P = 0.00003; P _FDR_ = 0.0007) showed a significant correlation with gout (**Figure 3**).

### 3.2 Sensitivity analysis

Due to the susceptibility of the IVW method to weak instrument bias, we conducted a sensitivity analysis on all potential correlations (**Supplementary File, Table S5**). In the Cochran’s Q test results, succinyl carnitine exhibited acceptable weak heterogeneity. After a pleiotropy analysis, we found that the MR Egger intercept test’s P-value for glycine was 0.032, indicating pleiotropy. Consulting the Phenoscanner V2 website (http://www.phenoscanner.medsci.cam.ac.uk/), we discovered that SNP rs715 is directly related to urate, leading to its exclusion. Post-exclusion, glycine’s IVW P-value changed to 0.363, suggesting no association with SU. No abnormalities were detected in pleiotropy tests of blood metabolites significantly linked to SU and gout. Removing any SNP in the “leave-one-out” method did not affect stability.

### 3.3 Reverse MR analysis

**Figure 4:**
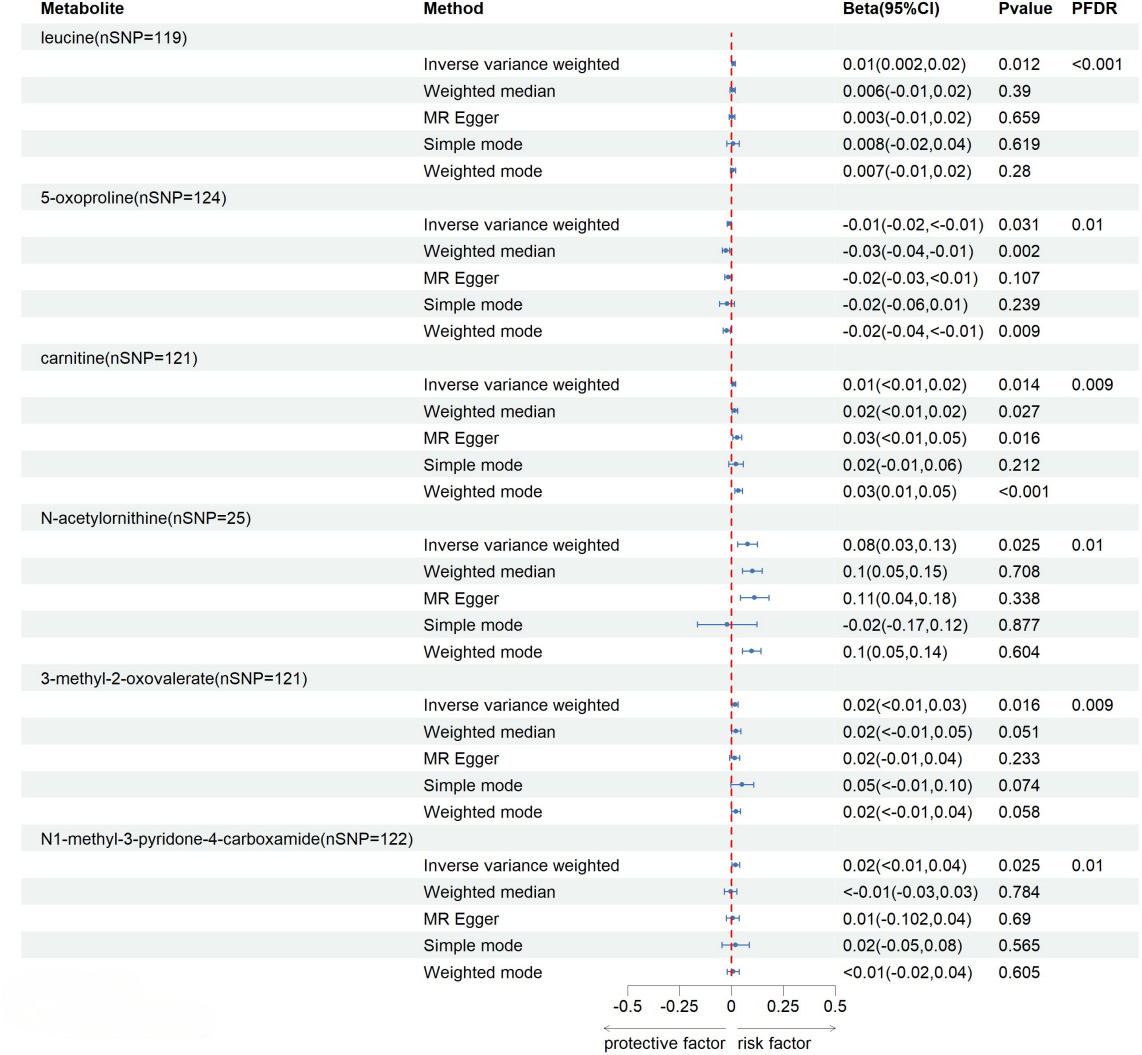
Relationship between serum urate and known blood metabolites after correction for FDR.

**Figure 5:**
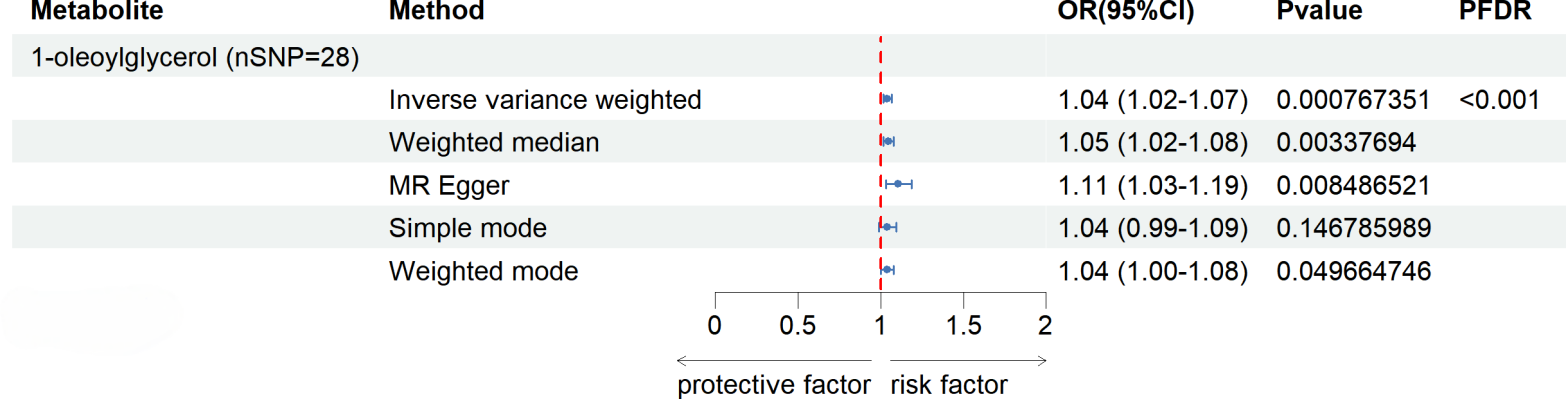
Relationship between gout and known blood metabolites after correction for FDR.

The reverse MR analysis indicated a significant association between SU and six kown blood metabolites, but their influence on SU is marginal (**Figure 4**). One kown metabolite exhibits a significant correlation with gout (**Figure 5**).

### 3.4 Metabolic pathway analysis

**Figure 6:**
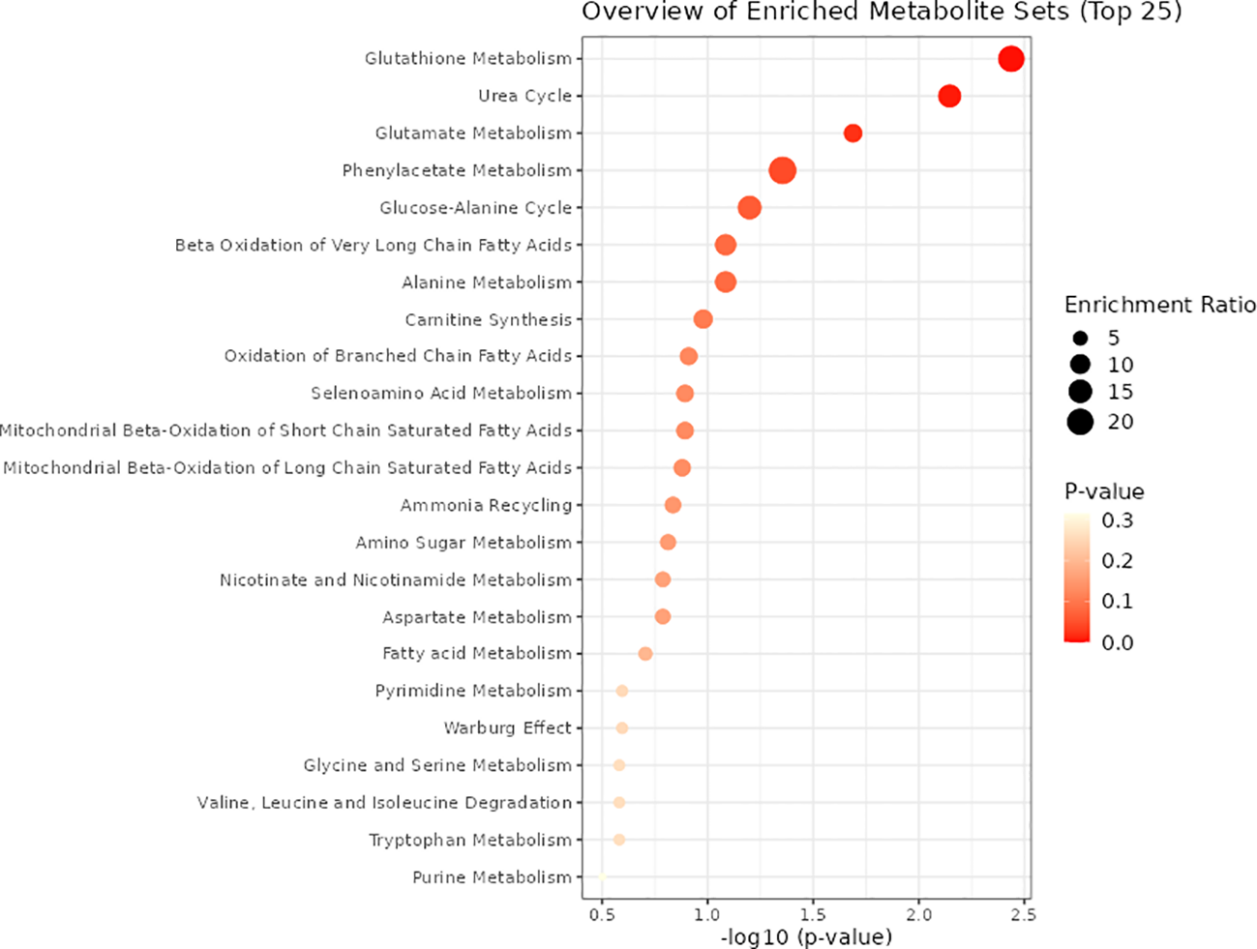
Metabolite KEGG enrichment analysis.

As illustrated in **Table 1**, four metabolic pathways exhibit associations with metabolite binding and SU. The most prominent among them is the Arginine biosynthesis pathway, followed by Alanine, aspartate, and glutamate metabolism, Nitrogen metabolism, and Valine, leucine, and isoleucine biosynthesis. Additionally, we conducted a metabolite enrichment analysis, unveiling significant enrichments in Glutathione Metabolism, Urea Cycle, Glutamate Metabolism, and Phenylacetate Metabolism (**Figure 6**).

**Table 1:** Metabolic pathways affecting serum urate.

Metabolic pathway	metabolites involved	P value	Database
Arginine biosynthesis	N-Acetylornithine; Glutamine	0.00072301	KEGG
Alanine, aspartate and glutamate metabolism	L-Alanine; L-Glutamine	0.0029499	KEGG
Nitrogen metabolism	L-Glutamine	0.018927	KEGG
Valine, leucine and isoleucine biosynthesis	L-Leucine	0.025172	KEGG

KEGG, Kyoto Encyclopedia of Genes and Genomes.

## 4 Discussion

In recent decades, metabolomics has played a crucial role in researching various diseases, leading to significant progress in understanding the pathogenesis and risk factors of hyperuricemia and gout. However, most studies have been confined to animal models or small case-control studies, limiting their ability to establish causal relationships.

In this study, we identified a total of 16 blood metabolites associated with SU concentration, with three linked to gout. Among the blood metabolites related to SU, four known blood metabolites showed a significant correlation after FDR correction, and one gout-related metabolite exhibited a significant association. These findings were robust across various testing methods. Additionally, we detected 4 metabolic pathways associated with SU concentration. Simultaneously, in the reverse MR analysis, we identified 7 blood metabolites significantly associated with SU levels, and one associated with gout. To the best of our knowledge, this marks the first systematic evaluation of the MR study on human blood metabolites and SU levels. Our research contributes to unraveling the role of human blood metabolites in the early diagnosis and prevention of gout and hyperuricemia, offering novel insights for future metabolomics studies. With the growing emphasis on understanding the relationship between blood metabolites and diseases in clinical settings, there has been continuous exploration of blood metabolites associated with SU concentration and gout. Hyperuricemia and gout are no longer perceived as isolated pathologies; their underlying mechanisms may be interconnected with various comorbidities. Numerous studies have linked hyperuricemia and gout to conditions such as renal disorders, diabetes, metabolic syndrome, and aging (12, 37). Currently, blood stands out as the most crucial and commonly used sample in metabolomics. The versatility of blood samples lies in their ability to reveal a myriad of detectable blood metabolites. Moreover, in large-scale studies, obtaining blood samples is often more feasible than other sample types. This accessibility forms the foundation for early screening of hyperuricemia and gout. Metabolomics has unveiled alterations in the metabolic profiles related to uric acid levels, including changes in amino acids (38), energy blood metabolites (39), and lipids (40). Our research not only validates the presence of these metabolic shifts in SU and gout but also identifies key blood metabolites and pathways associated with SU levels and gout pathogenesis.

Amino acids serve diverse functions within the human body, with a particular emphasis on their pivotal role in protein synthesis (41). Leucine, isoleucine, and valine collectively constitute the branched-chain amino acids (BCAAs). Previous studies have demonstrated that leucine is significantly altered in the course of several metabolic diseases (42). Additionally, in overweight and obese (OB) children, leucine concentrations were significantly elevated (43), showing a positive correlation with inflammatory markers (CRP, IL-6), SU, visceral adiposity, type 2 diabetes (T2D), insulin resistance, and CVD (38, 39). Simultaneously, levels of inflammation-related fibrinogen and hs-CRP displayed an increasing trend with elevated leucine levels (44). Given the limited sample size in previous studies, we cannot infer the precise mechanism underlying the correlation between leucine and IL-6. While IL-6, a standard inflammation marker (45), does not directly contribute to SU elevation, its levels increase upon activation of MSU (46). Nevertheless, the role of leucine in predicting SU and gout remains a topic of debate. A study discovered elevated valine levels in patients with acute gout and hyperuricemia, while the increases in leucine and phenylalanine were not statistically significant (47). However, the correlation between leucine and UA is likely due to abnormalities in glucose metabolism leading to disruptions in amino acid metabolism (48). Additionally, it may also be related to the dietary habits of the patients themselves (49). Hyperuricemia patients tend to consume more meat, eggs, and dairy products, and have higher intakes of fructose-containing beverages and alcohol compared to healthy individuals (50). These unhealthy lifestyle habits can lead to the development of metabolic syndrome and insulin resistance in the patients (51). In our study, we identified a robust correlation between the biosynthesis and degradation pathways of valine, leucine, and isoleucine and SU levels. In general, our research findings are in partial agreement with previous studies. Hence, conducting further in-depth research with larger sample sizes is imperative to elucidate the intricate relationship between leucine and SU.

The typical absence of N-acetylornithine expression in the human body stems from the non-expression of specific enzymes responsible for its processing in mammals. In microorganisms, N-acetylornithine primarily contributes to arginine synthesis (52), while in plants, its principal role lies in ornithine synthesis (53). The current literature on the correlation between N-acetylornithine and SU levels is limited, with a handful of studies suggesting a potential link to impaired kidney function. Uric acid, the final product of purine metabolism in the human body, is usually excreted through urine. Disruptions in uric acid metabolism occur as renal function declines, leading to abnormal SU levels. A GWAS assessing concentrations of 308 non-targeted blood metabolites in African Americans found an association between elevated N-acetylornithine levels and kidney function, as indicated by creatinine levels (54). Daily protein intake, particularly from plant sources, correlates with the progression of Chronic Kidney Disease (CKD) (55, 56). Metabolite sample testing in CKD patients post-kidney transplantation revealed a significant decrease in concentrations of key renal function indicators (creatinine, eGFR, cystatin C) with elevated N-acetylornithine levels (57). Our MR study found a significant bidirectional relationship between N-acetylornithine and SU, with both being risk factors for the other, so we speculate that the relationship between N-acetylornithine and SU may be mediated bi-directionally through renal function, and when one of these factors leads to impaired renal function, it affects the other. Given the close and pronounced relationship between N-acetylornithine and SU levels, it emerges as a promising biomarker. However, a more comprehensive understanding of its mechanistic implications requires further dedicated clinical investigations into the nuanced relationship between N-acetylornithine and SU levels.

Succinyl carnitine, a form of acylcarnitine, plays a pivotal role in mitochondria by facilitating the uptake of fatty acids, a crucial step in the β-oxidation process (58). Within the tricarboxylic acid cycle, succinyl coenzyme A undergoes conversion into succinyl carnitine, actively participating in various metabolic pathways. Recent studies have unveiled a correlation between succinyl carnitine levels and systemic inflammatory response as well as heart failure (59, 60). Our MR study unequivocally demonstrates a significant causal relationship between succinyl carnitine and elevated SU levels. Despite shedding light on their connection, the precise mechanism linking succinyl carnitine to SU elevation remains elusive. Thus, a pressing need exists for further experimental and clinical research to delve deeper into understanding the intricate relationship between these two factors.

The human body utilizes niacin (nicotinic acid, vitamin B3) or nicotinamide (NAM) along with dietary tryptophan as substrates to synthesize nicotinamide adenine dinucleotide (NAD). N-methyl-2-pyridone-5-carboxamide (2PY) and N1-methyl-4-pyridone-3-carboxamide (4py) are degradation products of NAD, recognized as uremic retention molecules eventually excreted through urine (61). Studies indicate that the excessive accumulation of 4py may inhibit the biological activity of PARP-1, adversely affecting uric acid production (62). Current research on 4py primarily focuses on kidney diseases, inflammatory responses, and diabetes. In a study on children with chronic renal failure (CRF), a positive correlation was found between 4py and creatinine concentration, while a negative correlation was observed with creatinine clearance. This suggests that the accumulation of 4py parallels renal dysfunction, possibly due to the decline in renal function in CRF patients (63). Research on systemic inflammatory response syndrome, measuring the metabolic profile of hemodialysis patients and its association with renal function, revealed a significant negative correlation between 4py and renal function as renal function declined (64). The established correlation between SUA concentration and renal function is supported by a retrospective cohort study assessing the association between SU levels and glomerular filtration rate (eGFR) in the Chinese population. Elevated SU was also significantly correlated with the risk of CKD (65). Another clinical study measuring the content of uracil and purines in the urine of patients at different stages of CKD found a positive correlation between GFR and the ratio of uric acid, xanthine, and hypoxanthine in urine (66). Although existing research cannot conclusively prove a direct impact of 4py on SU levels, there is reason to believe that there may be a genetic causal relationship between them. This connection may be mediated through the impact of 4py on renal function.

Findings from our study indicate that an elevated genetic susceptibility to increased levels of 1-oleoylglycerol is associated with a higher risk of gout. Previous research has established a close connection between disruption in lipid metabolism and gout risk (67). Currently, there is limited research on 1-oleoylglycerol. In a recent large-scale clinical study, the metabolic profiling of gut microbiota-related blood metabolites and their correlation with T2D was analyzed in 5,000 middle-aged Finnish men. After correcting for various factors such as smoking, age, BMI, and physical activity, it was found that 1-oleoylglycerol, controlled by the gut microbiota, increased the incidence of T2D by 29%, showing a significant association with the onset of T2D (68). Existing research has already confirmed that changes in the gut microbiota contribute to an increased risk of gout (69, 70). Given the gut microbiota’s potential to increase the risk of gout, 1-oleoylglycerol may induce gout through alterations in the gut microbiota. However, further exploration is required under specific experimental conditions to substantiate this hypothesis.

Our study boasts several strengths. Firstly, we systematically investigated 486 blood metabolites, providing the most comprehensive analysis to date of the metabolic characteristics associated with hyperuricemia and gout. Secondly, prior to conducting the MR study, we performed LDSC analysis to ensure a potential genetic correlation between the studied blood metabolites and both SU levels and the occurrence of gout. This precautionary step minimizes biases arising from polygenicity and confounding factors, thereby enhancing the robustness of our results. Lastly, adopting a bidirectional MR study approach, which considers both causal directions, helps mitigate potential issues related to reverse causation and residual confounding factors.

However, this study also has some limitations. Firstly, we utilized publicly available GWAS data, and potential sample overlap might introduce confounding bias. Secondly, our study’s exposures and outcomes are derived from the European population, warranting further investigation to determine whether similar causal relationships exist in other populations. Thirdly, the limited number of participants in our exposure dataset may result in missing relationships between blood metabolites and SU levels and gout. Additionally, some blood metabolites and pathways in this study lack a comprehensive understanding of their mechanisms and pathological relationships with SU and gout, limiting the interpretation of our MR analysis results. While MR studies contribute to identifying potential blood metabolites associated with SU and gout, further clinical and experimental research is essential to explore the correlations between them.

## 5 Conclusion

Two-sample MR studies unveiled the significant roles of five serum blood metabolites associated with SU levels and gout. Nineteen blood metabolites were identified with potential causal relationships with SU levels and gout, elucidating four crucial metabolic pathways in SU levels. The discoveries from this study aid in comprehending the biological mechanisms of hyperuricemia and gout, paving the way for future targeted therapeutic interventions.

## Data availability statement

The original contributions presented in the study are included in the article/**Supplementary Material**, further inquiries can be directed to the corresponding authors.

## Author contributions

YZ: Writing – original draft, Conceptualization, Data curation, Investigation, Methodology, Visualization. CAY: Writing – original draft, Investigation. BHZ: Data curation, Writing – original draft, Investigation. YWC: Investigation, Visualization, Writing – original draft. WJC: Writing – original draft. GCW: Writing – original draft. CWZ: Writing – review & editing. WHZ: Writing – review & editing.
